# Countermovement Jumps Detect Subtle Motor Deficits in People with Multiple Sclerosis below the Clinical Threshold

**DOI:** 10.3390/biomedicines11030774

**Published:** 2023-03-03

**Authors:** Anne Geßner, Heidi Stölzer-Hutsch, Katrin Trentzsch, Dirk Schriefer, Tjalf Ziemssen

**Affiliations:** Center of Clinical Neuroscience, Neurological Clinic, University Hospital Carl Gustav Carus, TU Dresden, Fetscherstr. 74, 01307 Dresden, Germany

**Keywords:** multiple sclerosis, motor deficits, assessment, jump analysis, countermovement jump

## Abstract

In the early stages of multiple sclerosis (MS), there are currently no sensitive assessments to evaluate complex motor functions. The countermovement jump (CMJ), a high-challenge task in form of a maximal vertical bipedal jump, has already been investigated as a reliable assessment in healthy subjects for lower extremity motor function. The aim was to investigate whether it is possible to use CMJ to identify subthreshold motor deficits in people with multiple sclerosis (pwMS). All participants (99 pwMS and 33 healthy controls) performed three maximal CMJs on a force plate. PwMS with full motor function and healthy controls (HC) did not differ significantly in age, disease duration, Body Mass Index and the Expanded Disability Scale Score. In comparison to HC, pwMS with full motor function demonstrated a significantly decreased CMJ performance in almost all observed kinetic, temporal and performance parameters (*p* < 0.05). With increasing disability in pwMS, it was also observed that jump performance decreased significantly. This study showed that the CMJ, as a high challenge task, could detect motor deficits in pwMS below the clinical threshold of careful neurological examination. Longitudinal studies are pending to evaluate whether the CMJ can be used as a standardized measure of disease progression.

## 1. Introduction

Multiple sclerosis (MS) is an inflammatory chronic disease of the central nervous system, which damages the myelin layer of the nerve fibers [1]. Therefore, early initiation of therapy is critical for a more favourable progression of disease [2]. MS often results in decreased motor functions, which may rely upon integrated involvement of neuromuscular, neurosensory, musculoskeletal, and cardiopulmonary systems [3]. Equally as complex as the motor system is the identification of subtle motor deficits in people with multiple sclerosis (pwMS).

In the early stage of disease in MS, when pwMS have no or minimal disability, there are currently no sensitive objective assessments to identify subtle motor deficits in the lower extremities as well as to identify and quantify early deficits in complex movements. However, the ability to identify, specify and monitor subtle motor impairments is critical to the management of optimal disease-modifying and symptomatic treatment [4]. In the early stages of MS, neurological reserve can functionally compensate for neuronal damage caused by the demyelinating lesions through various mechanisms involving, for example, cerebral reorganization [5,6]. Currently, the assessment of MS patients occurs as a neurological examination and on rare occasions by a standardized functional test.

Neurological examination is standardized using the Expanded Disability Status Scale (EDSS) [7].

Krieger et al. [8] reported in their study that traditional clinical measures (i.e., EDSS) did not distinguish neurologically “normal” pwMS (i.e., EDSS 0) from healthy controls (HC). High-challenge tasks were more sensitive to subtle deficits than traditional clinical measures in pwMS with an EDSS 0 and could detect subthreshold impairments and identify underlying disease burden [8]. Assessments such as the EDSS are quite subjective in evaluating the different functional systems separately [9]. In contrast, high-challenge tasks represent a complex integration of different functional systems (e.g., motor, cerebellar, sensory) requiring great physical effort.

The countermovement jump (CMJ) exemplifies a high-challenge task, challenging the neuromuscular system by combining strength, balance, coordination and muscle timing in one assessment. As a type of vertical jump, the CMJ works on the principle of the stretch-shortening cycle (SSC). The SSC is defined as a high-intensity eccentric contraction immediately before a rapid concentric contraction and occurs in natural movements such as walking and running [10].

The CMJ is commonly used in professional sports to assess lower-body ballistic performance and monitor the effectiveness of training programs [11,12,13]. Many research studies confirm the high validity and reliability of the CMJ on force plates to assess motor function of the lower extremity in a variety of clinical settings [12,14,15,16,17,18,19,20].

To our knowledge, there are no studies that investigate CMJ performance in MS. Two pilot studies from Kirkland et al. [21,22] provide the first evidence that jump tasks can detect differences between pwMS with mild disability and healthy controls and that they are a potentially useful measurement of lower limb function in pwMS. However, in contrast to our study, Kirkland et al. assessed the use of horizontal jumps on an instrumental walkway system with small sample size. The combination of multiple domains in one test enables the identification of deficits and facilitates adequate rehabilitation [4].

In this study, we created a MS group equal to HC according to the degree of disability, motor function assessed with EDSS and physical activity. We aimed first (i) to investigate the CMJ performance between pwMS groups and HC and second (ii) to evaluate the suitability of the CMJ for detecting motor deficits in pwMS below the clinical threshold of the pyramidal FSS. The clinical threshold is defined by clinical signs of an underlying burden of disease. Below the clinical threshold, clinical signs may not be apparent on routine clinical examination, and when lesions cross the clinical threshold, clinical symptoms occur [8]. The third aim (iii) was to assess the CMJ performance complementary to subthreshold EDSS measures in sensory and cerebellar FSS.

## 2. Materials and Methods

### 2.1. Participants

We conducted a cross-sectional study in the MS Center at the Center of Clinical Neuroscience of the Department of Neurology, University Hospital Carl Gustav Carus, Dresden, Germany. HC without neurological disease and pwMS were invited to participate. We recruited 189 subjects between April 2021 and September 2021. All participants provided written informed consent for the study. The study was approved by the local ethics committee (BO-EK-320062021).

Inclusion criteria were as follows: (a) presence of confirmed MS diagnosis according to McDonald’s criteria, (b) relapse-free status in the past 3 months, (c) EDSS Score between 0 and 5.0, (d) age between 18 and 65 years, (e) ability to walk without aid and rest for ≥500 m and (f) to perform heel rise, stand on heels and perform squats. Prior to jump testing, physical activity was assessed by patients using Godin Leisure Time Exercise Questionnaire (GLTEQ). Exclusion criteria were with the presence of orthopedic and surgical disorders that affect jumping, history of falls in the past month, fear of falling while jumping and current pregnancy. In all study participants, first the EDSS was recorded, followed by the GLTEQ and finally the CMJs.

To standardize physical performance, a GLTEQ-Score ≥ 24 provided the basis of participants included in the analysis. Further, for comparability in terms of motor skills between pwMS and HC, two groups were divided retrospectively in pwMS with normal (full) motor function (pwMS _motor normal_) and pwMS with impaired motor function (pwMS _motor impaired_). PwMS _motor normal_ were defined as having an EDSS score ≤ 1.5, with no motor abnormalities and disability appearing below this clinical threshold. As per the EDSS classification, an EDSS Score ≤ 1.5 resulted in pyramidal FSS of 0–1, normal muscle strength and unimpaired monopedal hopping. In all these items, the pwMS _motor normal_ were equal to the HC. The pwMS _motor impaired_ showed an EDSS score ≥ 2 and therefore a pyramidal FSS of 2–4, reduced muscle strength and impaired monopedal hopping. Hence, this group was above the clinical threshold. In a final step, to test the suitability of the CMJ, only pwMS _unimpaired_ and HC were compared. Notably, both groups showed normal results in the cerebellar and sensory functional systems which affect the neuromuscular control and motor function.

### 2.2. Assessments

#### 2.2.1. Expanded Disability Status Scale (EDSS)

To examine the clinical status, the EDSS was assessed by certified raters in pwMS including MS-specific pyramidal, cerebellar and sensory FSS [9]. The EDSS is the most used disability scale in MS and is well established among neurologists [23]. In this study, HC were also examined with the complete EDSS. Participants were classified according to Kurtzke [9] as follows:

(a) No disability in pyramidal FSS: pyramidal ≤ 1.
(b) Normal sensory and cerebellar function: sensory FSS = 0 and cerebellar FSS = 0.

As part of the EDSS in the pyramidal FSS, the British Medical Research Council Rating Scale (BMRC) was assessed for lower extremity muscles [24]. Participants were classified according to BMRC as follows:

(a) Participants with normal muscle strength: full strength in all assessed muscle groups of the lower extremity.
(b) Participants with reduced muscle strength: not full strength in one or more muscle groups of the lower extremity.

Monopedal hopping was also performed as a part of the pyramidal FSS in the EDSS. Participants were classified according to monopedal hopping as follows:

(a) Participants with monopedal hopping unimpaired: normal, 10 jumps performed on one leg right and left.
(b) Participants with monopedal hopping impaired: less than 10 jumps on one or both legs.

#### 2.2.2. Godin Leisure Time Exercise Questionnaire (GLTEQ)

The GLTEQ is a validated patient-reported outcome (PRO) for measuring simple and effective physical activity in pwMS [25]. It is a three-item questionnaire to record the frequency at which the subjects performed physically strenuous, moderate, and mild exercise per week in the past month. A GLTEQ score of less than 14 units indicates insufficient activity, 14 to 23 indicated moderate activity and 24 units or more indicated high activity [25].

#### 2.2.3. Countermovement Jump (CMJ)

**Figure 1:**
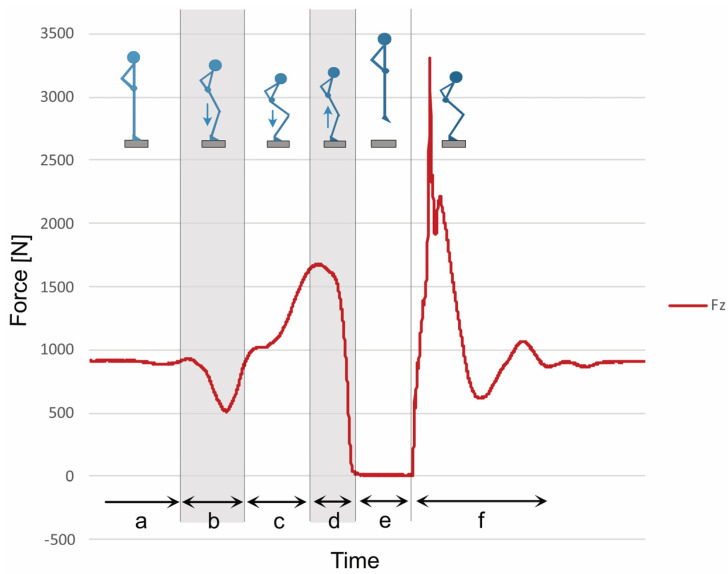
Countermovement jump phases in ground reaction curve. a = weight phase, b = unweight phase, c = eccentric phase, d = concentric phase, e = flight phase, f = landing phase; Fz = resultant force.

All participants performed three maximal CMJs without arm swing on a single force plate. Before the jumps, a physiotherapist verbally explained and demonstrated the jumping technique to each subject. The participants were instructed to jump as high as possible with their hands on their hips and to keep their legs extended during the flight phase of the jump (Figure 1). A 5 s rest was performed between the jumps, as described in previous studies [26,27,28]. Any CMJs that were inadvertently performed with the inclusion of arm swing or tucking of the legs during the flight phase of the jumps were excluded. All of the participants completed a practice jump before data collection. The jumping trials were performed wearing socks and everyday clothes.

The CMJ can be divided into 6 phases (Figure 1.) First (a), the patient stands still on the force plate and the body weight is measured. In the second phase (b), the patient begins a short countermovement with flexion of the hips and knees in which the body weight is reduced below a threshold value of 5%. The phase ends when the body weight in the force–time curve is reached again. Third is the braking or eccentric phase (c), characterized by flexion of the hip, knee, and ankle until the center of mass (COM) is lowest and velocity is zero. During the braking phase, the following leg muscles work eccentrically: M. gluteus maximus, M. iliopsoas, M. quadriceps femoris, and M. triceps surae. Next is the propulsion or concentric phase (d). It begins with a forceful extension of the hips, knees, and ankles to move COM upwards and push off the force plate. The muscles which previously, in the braking phase, worked eccentrically now work concentrically. The time after take-off from the force plate to the highest point of the COM is described as the flight phase (e). The CMJ ends with the landing phase (f) when both feet touch the force plate and the initial position is reached again [29].

### 2.3. Data Collection

Ground reaction forces and moments of force were recorded using a portable single force plate from AMTI (Advanced Mechanical Technology Inc., Watertown, MA, USA, AccuPower-O) and sampled at 1000 Hz. Force plates are the gold standard and a valid method of measuring vertical jump performance [30,31]. The reliability of the AMTI force plate is good to excellent and shows a low error rate of 2.5% [32].

A dedicated biomechanical analysis software (AccuPower Solutions, Version 1.5.4.2082, Watertown, MA, USA) was used to record the parameters during the different jump phases. The most common and reliable jump parameters in sports medicine depending on the different CMJ phases were selected [33]. An important consideration in the extraction of the jump parameter was to analyse not only force parameters, but also time-based parameters, as these are more indicative of neuromuscular performance [14,34]. Table 1 shows a description of the recorded jump parameters.

### 2.4. Statistical Analysis

For all three jumps, the mean values of the individual parameters were used for the statistical analyses. Force values were converted to values relative to body mass. The distribution of all jump parameters was visually inspected and supplemented with the Shapiro–Wilk test for the assessment of normality.

In the evaluation of jump parameters, a descriptive specification of mean values and standard deviations occurred. Generalized linear mixed models (GLMM) were applied to determine the differences in jump parameters between the groups (HC, pwMS _motor normal_ and pwMS _motor impaired_) and subgroups according to normal cerebellar and sensory FSS (HC and pwMS _unimpaired_) adjusted for age, gender and Body Mass Index (BMI). For normally distributed outcomes, the Gaussian distribution with identity link was used, while for right-skewed outcomes, the Gamma distribution with log link function was used. Statistical significance was fixed at *p* < 0.05. The significance level α was Bonferroni corrected for multiple testing. Effect sizes in between-group comparisons were quantified using Cohen’s d, with effect sizes defined as small (d = 0.20–0.49), moderate (d = 0.50–0.79) or large (d > 0.80) [35]. Spearman rank correlations were calculated to study bivariate relations of jump parameters with EDSS and FSS. Statistical analyses were performed using IBM Statistical Package for the Social Sciences (SPSS) for Windows, Version 28 (IBM Corp, Armonk, NY, USA).

## 3. Results

### 3.1. Participants

**Figure 2:**
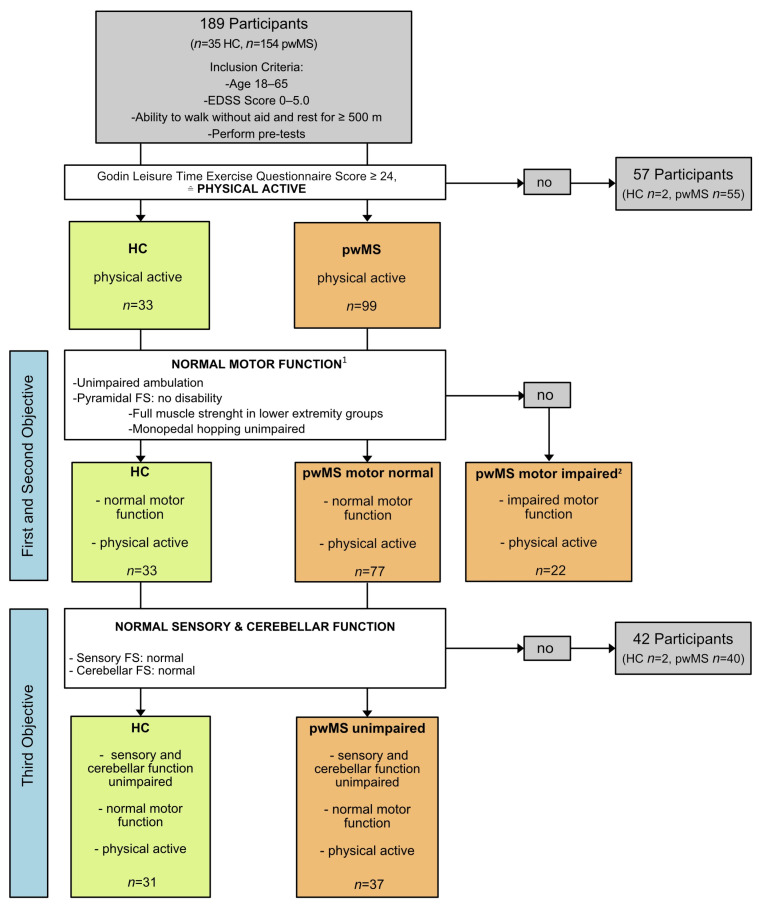
Flowchart of the study population. In order to create the best possible basis for comparability between groups, only participants with a GLTEQ ≥ 24, i.e., physically active, were included in the analysis. In a next adjustment step, the group of pwMS was divided according to the degree of disability and motor impairment assessed with EDSS. This resulted in a pwMS group with motor disability (pwMS _motor impaired_) and a pwMS group without motor disability (pwMS _motor normal_). PwMS _motor normal_ thus corresponds to the HC group (all participants were examined with EDSS). In this study, the jumping performances between these three groups were analysed in two steps (first and second objective). Because sensory and cerebellar dysfunction can influence motor function, only participants without sensory and cerebellar dysfunction according to the EDSS were included in a finally analysis step (tertiary objective). ^1^ Normal motor function: EDSS score ≤ 1.5 with pyramidal FSS ≤ 1. ^2^ PwMS motor impaired: EDSS score ≥ 2 with pyramidal FSS of 2–4, reduced muscle strength and impaired monopedal hopping. Abbreviation: EDSS = Expanded Disability Status Scale; GLTEQ = Godin Leisure Time Exercise Questionnaire; FSS = functional system score; pwMS = people with multiple sclerosis; HC = healthy controls.

A total of 189 study participants were examined in the study. After evaluation of the GLTEQ, *n* = 132 study participants emerged as physically active and were therefore included in the analysis (Figure 2). PwMS _motor normal_ and HC are equal regarding to degree of disability, motor function according to EDSS and physical activity and did not differ based on age, BMI or gender (Table 2). The pwMS _motor impaired_ had a significant higher age (*p* < 0.05), EDSS score (*p* < 0.001) as well as pyramidal FSS, sensory FSS and cerebellar FSS (*p* < 0.001), as expected. An overview of the participants’ characteristics is shown in Table 2.

### 3.2. Group Comparison between MS Groups and HC in CMJ Performance

**Figure 3:**
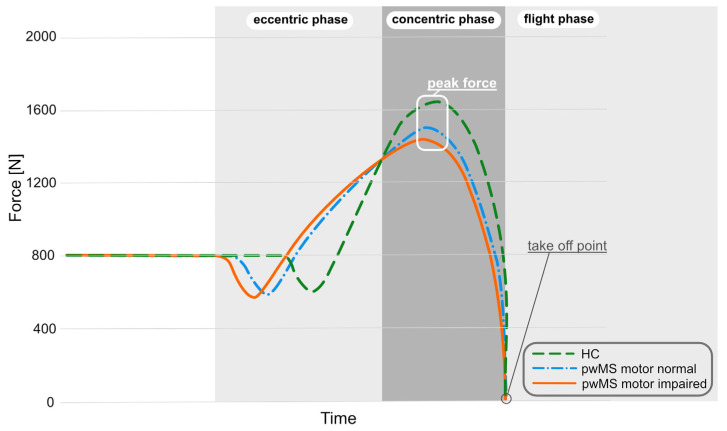
Examples of the countermovement jump force–time curves during the contraction time for pwMS _motor normal_, pwMS _motor impaired_, and HC. HC shows a rapid increase in force in the eccentric phase and a higher peak force than pwMS. Compared to HC and pwMS _motor normal_, the pwMS _motor impaired_ show the longest contraction phase (eccentric + concentric phase). Abbreviations: HC = healthy controls; pwMS = people with multiple sclerosis.

Significant differences between the three groups could be observed in almost all jump parameters, except jump height (Table 3). PwMS _motor normal_ showed a better jump performance than pwMS _motor impaired_ and HC better than pwMS _motor normal_ (pwMS _motor impaired_ < pwMS _motor normal_ < HC). Significant differences were detected between the MS groups regarding to temporal parameters (except braking time), kinetic parameters (except peak force) and performance parameter. PwMS _motor impaired_ differed significantly in all jump parameters from the HC. The comparison of examples of the CMJ force–time curves during the contraction time between pwMS _motor normal_, pwMS _motor impaired_ and HC is shown in Figure 3.

### 3.3. Group Comparision between pwMS with Normal Motor Function and HC

Significant differences between the HC and pwMS _motor normal_ could be observed in all kinetic and temporal parameters (except propulsive time) (Table 3). PwMS _motor normal_ showed a significantly decreased eccentric and concentric force, resulting in significantly shorter flight time. A significant imbalance in the ratio between flight time and contraction time as well as between braking impulse and propulsive impulse could be observed for pwMS _motor normal_ in comparison to HC. Small effect sizes were detected for all jump parameters. The largest effect sizes were shown for flight time (d = 0.461), average negative power (d = 0.404) and braking time (d = 0.358).

### 3.4. Group Comparision between HC and pwMS with Full Motor, Sensory and Cerebellar Function

**Figure 4:**
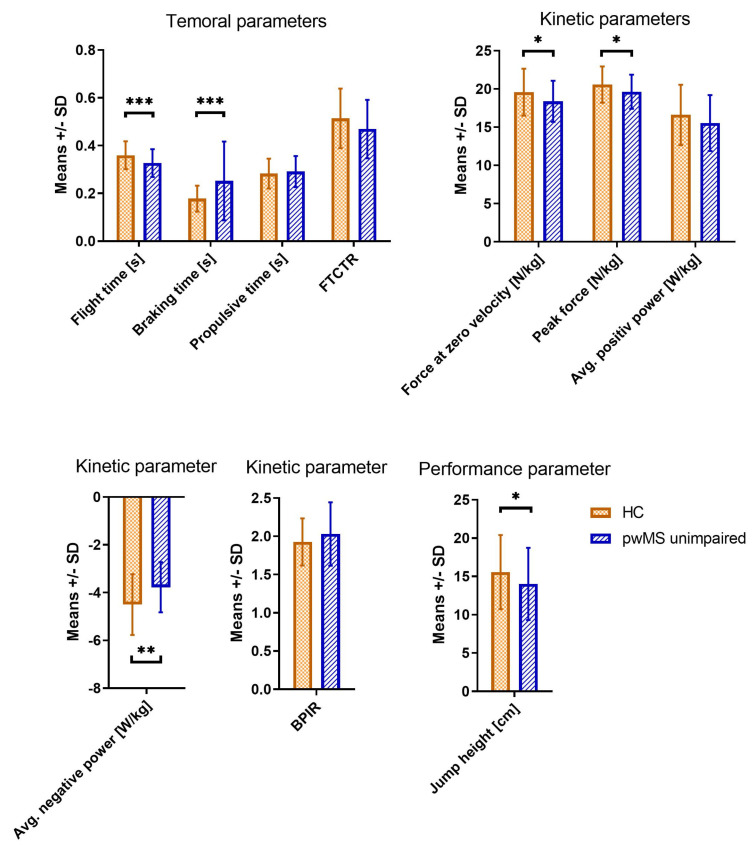
Group comparison between pwMS _unimpaired_ and HC according to normal sensory and cerebellar FSS for temporal, kinetic and performance jump parameters. Data presented as mean with standard deviation and significance indicators (*** *p* ≤ 0.001; ** *p* < 0.01; * *p* < 0.05). Abbreviations: HC = healthy controls; pwMS = people with multiple sclerosis; FTCTR = flight time to contraction time ratio; Avg. = average; BPIR= brake to propulsive impulse ratio.

Significant group differences in jumping performance between pwMS _unimpaired_ and HC, both with normal sensory and cerebellar function, were observed in the eccentric phase (braking time, force at zero velocity, average negative power), flight phase (flight time, jump height) and peak force of the CMJ (see Figure 4). The highest effect size, with small effects for sensory and cerebellar FSS of 0, was detected for the same parameters as in the group comparison between pwMS _motor normal_ and HC.

### 3.5. Correlation of Jump Parameters According to EDSS

Overall, the bivariate comparison of the jump parameters and clinical outcome scores (EDSS) showed mild to moderate association (Table 4). The highest correlation coefficients were detected between kinetic parameters and EDSS with FSS. All jump parameters showed significant correlation with pyramidal FSS, but the highest correlation coefficients were obtained for the parameter average positive power and average negative power. The highest correlation was detected between cerebellar FSS and kinetic parameters from the eccentric phase of CMJ (force at zero velocity and average negative power). Similarly, the highest correlation for sensory FSS was also detected for average negative power.

## 4. Discussion

This study aimed to investigate the CMJ performance between pwMS groups and HC, as well as to evaluate the suitability of the CMJ on a force plate for detecting motor deficits in pwMS below different thresholds of neurological examination as part of EDSS. To our knowledge, this study is the first description of CMJ performance in pwMS.

First, we were able to create a MS group (pwMS _motor normal_) equal to HC according to the degree of disability, motor function assessed with EDSS and physical activity. We determined that pwMS showed decreased CMJ performances in comparison to HC. In comparison of the motor normal group of pwMS, it was shown that CMJ performance decreased in all kinetic and temporal parameters (except propulsive time) compared to HC. With increasing disability in pwMS, it was also observed that jump performance decreased significantly. Furthermore, the CMJ could detect significant deficits in flight time, peak force and eccentric-based parameter (braking time, force at zero velocity and average negative power) for pwMS _motor normal_ with additionally normal cerebellar and sensory function.

As we assumed, our results suggest that pwMS without obvious strength, coordination and sensory abnormalities of the legs require a more challenging task such as the CMJ to demonstrate motor deficits below the clinical threshold of the EDSS. These results confirm the findings of Krieger et al. [8] and suggest that pwMS, with full muscle strength, normal cerebellar and sensory function as assessed by EDSS, indeed demonstrate motor impairments. The correlation results suggest that jump parameters are appropriate outcome measures indicating disability deterioration in pwMS, especially disability in motor function. In addition, the jump parameters could supplement the EDSS with sensorimotor and neuromuscular outcomes as metric variables.

Our study findings are consistent with Kirkland et al. in showing that bipedal hopping can detect and monitor sensorimotor control in pwMS who do not currently experience clinical deficits [4]. Compared to Kirkland et al., we used the CMJ instead of horizontal jumps as it is already a reliable and valid measure of muscle strength and neuromuscular control in sports medicine [14,36]. Furthermore, vertical jumps require less space and, with appropriate upholstering, can be performed safely.

We determined that CMJ performances in pwMS are characterized by a significantly lower force at zero velocity in the eccentric phase, in which participants squat to the lowest COM. Overall, the braking phase was significantly longer in pwMS (pwMS _motor normal_: +39%; pwMS _motor impaired_: +67%) than in HC (see Figure 3). To achieve a rapid change between eccentric and concentric phases, a high eccentric force is necessary to develop an even higher concentric force [29]. The low eccentric force in pwMS is therefore followed by a significantly lower peak force compared to HC (see Figure 3). This suggests a reduction in the strength of the lower limbs in pwMS, even in those with normal muscle strength according to the BMRC scale. In the eccentric to concentric phase ratio, pwMS demonstrated an inefficient SSC, as indicated by an increased BPIR compared to HC.

These findings have clinical relevance in the care of pwMS. Identifying early sensorimotor deficits is important to initiate early rehabilitative intervention [4,37]. The CMJ in MS can be useful not only for early detection but also for determining the exact movement impairment. By measuring multiple domains (coordination, balance, proprioception and strength) of impairment in one test, complex movement functions can be assessed, specific deficits can be identified and finally appropriate rehabilitation can be performed. For clinical practice, besides the force plate as the gold standard, there are also other possibilities such as video analysis, IPhone apps (“My Jump”) and optical-based systems to objectively assess CMJ performance [4,26].

Compared to the BMRC muscle strength assessment, which is part of the EDSS and tests only isolating concentric muscle strength, the CMJ measures the functional activity of the total muscle chain of the lower extremity. This functional assessment of muscle activity provides a better simulation of everyday movements as it is based on the principal of the slow stretch shortening cycle that occurs in natural movements such as walking and running [38].

Our study has the limitations of a cross-sectional study. Future studies with repeated measurements should be conducted to investigate whether the CMJ can be used as a standardized measure for the mid- and long-term assessment of disease progression and treatment response in MS. Furthermore, pwMS with a very low degree of disability, especially without limitation in the motor area according to EDSS, were examined; therefore, the EDSS total scores were very low, but not zero. To increase objectivity, isolated strength measurements should be included in future studies.

## 5. Conclusions

Our study is the first to provide evidence that the CMJ on a force plate, as a new assessment tool in MS, appears to be able to detect early motor and neuromuscular deficits in pwMS who have normal motor, cerebellar and sensory FSS according to the EDSS. Using the CMJ as a part of motor diagnosis, the deficits of eccentric and concentric muscle activity can be determined. These findings are useful in facilitating early and detailed rehabilitation approaches. The CMJ on a force plate as an objective and sensitive assessment could be a subthreshold test complementary to the neurological EDSS in the early stages of MS.

## Figures and Tables

**Table 1:** Measured jump parameters by force plate.

Jump Parameters	Description	Interpretation
Temporal parameters		
Flight time (s)	time in the air from jump take-off to landing	longer = better
Braking time (s)	duration of the eccentric phase	shorter = better
Propulsive time (s)	duration of the concentric phase	shorter = better
FTCTR	ratio of flight to contraction time	higher = better
Kinetic parameters		
FZV (N/kg)	maximum force during eccentric phase	higher = better
Peak force (N/kg)	maximum force during concentric phase	higher = better
ANP (W/kg) APP (W/kg)	average power during eccentric phase average power during concentric phase	higher = better higher = better
BPIR	ratio of braking to propulsive impulse	lower = better
Performance parameter		
Jump height (cm)	jump height calculated by force impact	higher = better

Abbreviations: FTCTR = flight time to contraction time ratio; FZV = force at zero velocity; ANP = average negative power; APP = average positive power; BPIR = brake to propulsive impulse ratio.

**Table 2:** Baseline characteristics of the study population. Data presented as mean (±standard deviation) unless specified otherwise.

	HC (*n* = 33)	pwMS Motor Normal (*n* = 77)	pwMS Motor Impaired (*n* = 22)
Age (years)	34.82 (±9.68) ^a^	35.86 (±8.83) ^a^	41.91 (±10.63)
Gender (female) *n* (%)	21 (63.6%)	54 (70.1%)	16 (72.7%)
Disease duration (years)	n.a	7.05 (±5.86)	9.27 (±6.63)
MS Subtype			
RRMS (%)	n.a	100%	100%
BMI EDSS (median, IQR)	24.95 (±4.97) 1.0 (0–1.0) ^a^	24.58 (±4.26) 1.5 (1.0–1.5) ^a^	25.63 (±5.26) 3.0 (2.5–3.5)
Pyramidal FSS	1.0 (0–1.0) ^a^	1.0 (1.0–1.0) ^a^	2.0 (2.0–2.5)
Cerebellar FSS	0 (0–0) ^a^	0 (0–1.0) ^a^	1.0 (1.0–2.0)
Sensory FSS	0 (0–0) ^a^	0 (0–1.0) ^a^	1.5 (1.0–2.0)

Abbreviations: pwMS = people with multiple sclerosis; HC = healthy controls; RRMS = relapsing-remitting multiple sclerosis; BMI= Body Mass Index; EDSS = Expanded Disability Status Scale; FSS = functional system score; IQR = interquartile range. ^a^ significant difference from motor impaired pwMS (*p* < 0.05).

**Table 3:** Jump parameters in pwMS and HC.

Jump Parameters	HC (*n* = 33)	pwMS Motor Normal (*n* = 77)	pwMS Motor Impaired (*n* = 22)	F (2,126)	*p*-Value
**Temporal parameters**					
Flight time (s)	0.36 ± 0.06 ^b,c^	0.32 ± 0.05 ^a,c^	0.27 ± 0.06 ^a,b^	22.24	<0.001 *
Braking time (s)	0.18 ± 0.05 ^b,c^	0.25 ± 0.15 ^a^	0.30 ± 0.19 ^a^	9.49	<0.001 *
Propulsive time (s)	0.28 ± 0.06 ^c^	0.30 ± 0.07 ^c^	0.43 ± 0.19 ^a,b^	10.57	<0.001 *
FTCTR	0.51 ± 0.12 ^b,c^	0.45 ± 0.11 ^a,c^	0.34 ± 0.12 ^a,b^	11.86	<0.001 *
**Kinetic parameters**					
FZV (N/kg)	19.61 ± 3.0 ^b,c^	17.52 ± 2.93 ^a,c^	14.99 ± 3.63 ^a,b^	11.43	<0.001 *
Peak force (N/kg)	20.57 ± 2.30 ^b,c^	19.34 ± 2.18 ^a^	17.92 ± 2.69 ^a^	5.711	0.004 *
ANP (W/kg)	−4.53 ± 3.86 ^b,c^	−3.41 ± 1.07 ^a,c^	−2.29 ± 0.89 ^a,b^	10.66	<0.001 *
APP (W/kg) BPIR	16.38 ± 3.86 ^b,c^ 1.92 ± 0.31 ^b,c^	15.08 ± 3.22 ^a,c^ 2.29 ± 1.14 ^a,c^	11.51 ± 3.76 ^a,b^ 2.29 ± 1.14 ^a,b^	25.56 13.71	<0.001 * <0.001 *
**Performance parameter**					
Jump height (cm)	15.38 ± 4.78 ^c^	13.73 ± 4.28	11.03 ± 5.22 ^a^	3.07	0.050

Data presented as mean (± standard deviation) unless specified. Abbreviations: HC = healthy controls; pwMS = people with multiple sclerosis; FTCTR = flight time to contraction time ratio; FZV = force at zero velocity; ANP = average negative power; APP = average positive power; BPIR = brake to propulsive impulse ratio; * = significant (*p* < 0.05). ^a^ significant difference with healthy group (*p* < 0.05). ^b^ significant difference with MS motor normal (*p* < 0.05). ^c^ significant difference with MS motor impaired (*p* < 0.05).

**Table 4:** Correlation between jump parameters and EDSS including pyramidal, cerebellar and sensory FSS in pwMS (*n* = 99) according to Spearman.

Jump Parameters	EDSS	Pyramidal FSS	Cerebellar FSS	Sensory FSS
**Temporal parameters**				
Flight time (s)	−0.295 **	−0.382 **	−0.263 **	−0.248 **
Braking time (s)	0.125	0.290 **	0.149	−0.68
Propulsive time (s)	0.225 *	0.267 **	0.337 **	0.143
FTCTR	−0.338 **	−0.391 **	−0.405 **	−0.188
**Kinetic parameters**				
FZV (N/kg)	−0.299 **	−0.336 **	−0.417 **	−0.239 *
Peak force (N/kg)	−0.249 *	−0.305 **	−0.348 **	−0.163
ANP (W/kg)	0.385 **	0.421 **	0.408 **	0.354**
APP (W/kg) BPIR	−0.374 ** 0.274	−0.440 ** 0.283 **	−0.358 ** 0.314 **	−0.203 * 0.325 **
**Performance parameter**				
Jump height (cm)	−0.248 *	−0.275 **	−0.108	−0.166

Abbreviations: FSS = functional system score; FTCTR = flight time to contraction time ratio; FZV = force at zero velocity; ANP = average negative power; APP = average positive power; BPIR = brake to propulsive impulse ratio; EDSS = Expanded Disability Status Scale; ** = *p* < 0.001, * = *p* < 0.05.

## Data Availability

All data produced in the present study are available upon reasonable request to the authors.
